# Preliminary inconclusive results of a randomised double blinded cross-over pilot trial in long-term-care dwelling elderly assessing the feasibility of stochastic resonance whole-body vibration

**DOI:** 10.1186/s11556-015-0150-y

**Published:** 2015-10-09

**Authors:** Slavko Rogan, Lorenz Radlinger, Dietmar Schmidtbleicher, Rob A. de Bie, Eling D. de Bruin

**Affiliations:** Department Health, Bern University of Applied Sciences, Bern, Switzerland; Department of Sport Science, Wolfgang-Goethe University Frankfurt, Frankfurt, Germany; Department of Epidemiology, CAPHRI School for Public Health and Primary Care, Maastricht University, PO Box 616, 6200 MD Maastricht, The Netherlands; Centre for Evidence Based Physiotherapy, Maastricht University, PO Box 616, 6200 MD Maastricht, The Netherlands; Department of Health Sciences and Technology, Institute of Human Movement Sciences and Sport, ETH Zurich, Wolfgang-Pauli-Str. 27, HIT J 31.2, CH-8093, Zurich, Switzerland

## Abstract

**Background:**

This randomised double-blinded controlled cross-over pilot study examined feasibility and preliminary effects of stochastic resonance whole-body vibration training applied in long term care elderly.

**Findings:**

Nine long term care elderly were recruited and randomized to group A (6 Hz, Noise 4 SR-WBV/ Sham) or B (Sham / 1 Hz, Noise 1 SR-WBV). Feasibility outcomes included recruitment rate, attrition, adherence and safety. Physical performance outcomes focused on the Expanded Timed Get Up-and-Go (ETGUG) test, the Short Physical Performance Battery (SPPB), and lower extremity muscle strength.

Of 24 subjects initially approached 9 started and 5 completed the study resulting in 37.5 recruitment, 44.4 attrition and 81.7 % adherence rates. No adverse events were reported. There is more evidence of improved performance levels in the SR-WBV treatment group with significant differences in average change for isometric rate of force development (*p* = 0.016 left leg; *p* = 0.028 right leg). No statistical significance was reached for other parameters.

**Conclusions:**

The findings of this study indicate that the used training protocol for long term care elderly is feasible, however, requires more closely monitoring of participants; e.g. needs protocol modifications that target improved compliance with the intervention in this setting. SR-WBV shows beneficial effects on physical performance for those adhering to the intervention.

**Trial registration:**

U.S. National Institutes of Health NCT01543243

**Electronic supplementary material:**

The online version of this article (doi:10.1186/s11556-015-0150-y) contains supplementary material, which is available to authorized users.

Physical activity (PA) for elderly is one of the major elements for general health prevention [1] and inactive or sedentary elderly should increase their PA [2]. Despite the known benefits of PA, residents living in long-term care (LTC) are relatively sedentary [3, 4]. Low baseline fitness and mobility levels in (pre-)frail elderly should be considered when starting exercise and this exercise should be adapted to the physical capabilities of these individuals [5].

Whole body vibration (WBV) seems a safe and beneficial type of balance exercise [6, 7]. Pilot studies showed that stochastic resonance WBV (SR-WBV) in (untrained) elderly is both safe and feasible [8, 9]. SR-WBV might also be valuable for (pre-)frail elderly in LTC where the neuromuscular systems of the trainees might not be able withstanding higher loading and long training sessions [8]. However, confirmatory results of such positive effects of WBV in LTC settings is not available and no evidence concerning the feasibility of SR-WBV in LTC dwelling elderly exists.

This study tested the feasibility and effects of SR-WBV training in LTC elderly with the aim to (I) evaluate the intervention process and the ability to recruit and retain LTC elderly for such an intervention, and (II) assess the impact of 4-week SR-WBV on physical performance.

## Findings

### Design

Nine LTC elderly (88.5 ± 6 years; height: 168 ± 1 cm; weight: 68.8 ± 14.3 kg) from “Senevita Residenz Multengut, LTC division Muri, Switzerland” volunteered in this randomised double blind controlled cross-over pilot study. Stratified by sex, participants were randomly assigned to group A or B by means of sealed opaque envelopes distributed after baseline assessments. Following Ethical Committee (Canton Berne) approval, informed consent was obtained prior to training (ClinicalTrial.gov: NCT01543243).

Inclusion criteria were aged over 65 years, being able to stand with or without aids, being classified as Resident Assessment Instrument (RAI [10]) performance level > 0, scoring > 22 points on the Mini-Mental Status Examination.

### Protocol

**Fig. 1:**
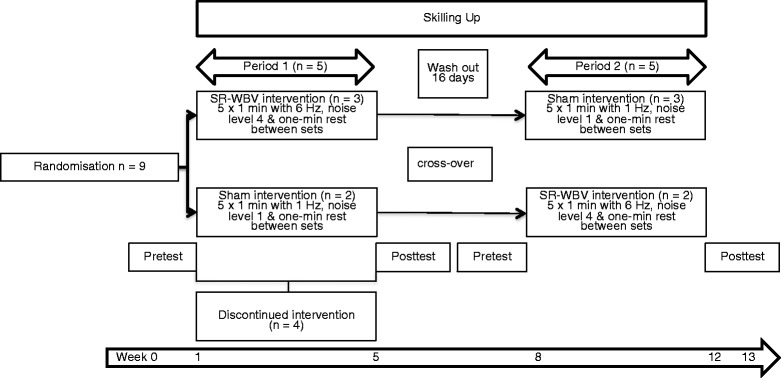
Difference values from period 1 - period 2. *P*-values were computed using Wilcoxon signed rank test for group 1 and group 2 at period 1 and period 2 *ES* effect size, *AP* anterior-posterior, *ML* medial-lateral, *FRT* Functional Reach Test, *ETGUG* Expanded Timed Get Up-and-Go, *ST* single task, *DT* dual task, *RTH* reaction time hand, *RTF* reaction time foot, *mm* millimetre, *s* seconds, *m/s* metre/seconds, *ms* milliseconds, *ss* sit-to-stand, m: metre

Participants were exposed to SR-WBV using a Zeptor med® device (Frei Swiss AG, Zurich, Switzerland). Participants stood freely on both legs wearing comfortable shoes with slight flexion of the hips, knees and ankle joints. In period 1, participants in group A received 5 sets of 1 min SR-WBV with 6 Hz, Noise 4 with 1 min of rest between sets, three times a week, during four weeks. One day rest between training sessions was warranted. Group B received a sham intervention of 5 sets of 1 min SR-WBV with 1 Hz, Noise 1. The 1 Hz frequency is expected to cause no training effect [8]. After a wash-out period of 16 days, treatment cross-over took place (Fig. 1). Secondary outcomes were scheduled at baseline (T0) before training, after four weeks training (T1) in period 1, and after four weeks training in period 2.

### Recruitment rate, rate of loss, program adherence

The criteria for success were based on the primary feasibility objective focussing on recruitment, attrition and adherence to the intervention [11]. Recruitment of a third of the residents deemed eligible, a 15 attrition rate, and 70 % training attendance rate were deemed acceptable [12].

### Secondary outcomes

The reliable Expanded Timed Get Up-and-Go (ETGUG) assessment measured time series of functionally important tasks [13] with details of the protocol reported elsewhere [8]. Physical performance was assessed with the short physical performance battery (SPPB), valid and reliable for lower extremity functions [14] and predictive for disability [15]. A dynamometer tested maximum isometric force (IMVC) and isometric rate of force development (IRFD) of the knee extensors (3 trials of testing were averaged to improve reliability [16]). IRFD was determined from the force-time curve calculating the steepness between the time points of 25 and 75 % IMVC. Details of the protocol were previously published [16].

### Statistical analysis

For recruitment, data for the total sampling frame for inclusion in the trial were taken. For the inclusion rate—i.e. the proportion of participants invited to participate who enrolled into the study—we distinguished between those who refused, did not respond or who were willing but excluded. Compliance to the treatment protocol was calculated using “Number of vibration sessions ÷ the total number of possible vibration sessions × 100”. Nonparametric Rank-Order Tests of Puri and Sen L Statistics [17] assessed change over time. Effect sizes (ES) were calculated for the differences within and between the groups and expressed as r = Z/√N; where r 0.1 is considered a “small”, 0.3 a “medium” and ≥0.5, a ‘large’ effect [18].

### Recruitment, attrition, and adherence

Tweenty four residents were deemed eligible to participate based on the inclusion–exclusion criteria and invited to participate. Nine LTC elderly individuals consented to partake resulting in 37.5 % recruitment rate. Fifteen participants declined with reasons given: no interest (*n* = 8), no motivation (*n* = 6) and personal reasons (*n* = 1). Five participants finished both study periods (age: 85.3 ± 6 years; height: 161.4 ± 6.2 cm; weight: 72.9 ± 15.9 kg) (Additional file 1) resulting in an attrition rate of 44.4 % (*n* = 4 drop-outs). The reasons for dropping out were: not motivated to continue (*n* = 3), and heart disease (*n* = 1). Adherence to the intervention for those continuing with training resulted in 81.7 % (98 of 120 training sessions) compliance.

### Secondary outcomes

Table 1 presents the test results and Table 2 summarises the intervention effects with ES per group for both periods. There are no carry-over effects for any of the outcome measures (Table 1), however, within group effect size for the SPPB (p = 0.039) is large. Between group effects with large ES are seen for the ETGUG 0 to 2 m, ETGUG 2 to 8 m, IRFD left and IRFD right.

**Table 1:** Repeated measures analyses of variance for ranked data for cross-over effects

	Pillai`s trace (r^2^ = SS_Bet_/SS_Tot_)	L [(N-1) r^2^]	Probability
ETGUG ss	0.208	0.789	0.440
ETGUG 0–2 m	0.536	3.462	0.160
ETGUG 2–8 m	0.931	4,467	0.332
ETGUG turn	0.833	5.000	0.167
ETGUG 12–18 m	0.833	5.000	0.167
ETGUG 18–20 m	0.556	1,250	0.444
ETGUG total time	0.833	5.000	0.167
SPPB	0.932	4.559	0.329
IMVC, left	0.556	3.750	0.148
IMVC, right	0.556	1.250	0.444
IRFD, left	0.772	2.600	0.278
IRFD, right	0.893	2.778	0.409

*ETGUG* Expanded Timed Get Up-and-Go, *IMVC* isometric maximal voluntary contraction, *IRFD* isometric rate of force development, *SPPB* Short Physical Performance Battery, *ss* sit to stand movement

**Table 2:** Results for physical performance within and between SR-WBV and Sham intervention

	SR-WBV (*n* = 5) Median (IQ)	*p* Within intervention	Sham (*n* = 5)	*p* Within intervention	*p* Between intervention
		ES	Median (IQ)	ES	ES
SPPB					
pretest	5.0 (3 – 5.5)	0.039*	6.0 (4.5 – 7)	0.10	0.396
posttest	6.0 (4.5 – 8)	0.92	6.0 (2.5 – 6.5)	0.73	0.27
ETGUG ss, (s)					
pretest	1.9 (1.2 – 2.7)	0.080	1.5 (0.9 – 2.0)	0.080	0.421
posttest	1.6 (0.9 – 2.0)	0.79	1.8 (1.3 – 3.3)	0.78	0.30
ETGUG 0–2 m (s)					
pretest	3.0 (2.4 – 3.9)	0.225	2.5 (2.1 – 2.9)	0.138	0.117
posttest	2.4 (2.1 – 3.0)	0.54	3.2 (2.3 – 3.9)	0.67	0.50
ETGUG 2–8 m, (s)					
pretest	8.3 (6.8 – 12.0)	0.043*	7.4 (6.0 – 8.0)	0.043*	0.009*
posttest	7.0 (6.0 – 7.8)	0.91	8.8 (8.2 – 9.9)	0.91	0.83
ETGUG turn, (s)					
pretest	9.5 (8.2 – 12.9)	0.043*	8.4 (6.9 – 9.3)	0.042*	0.402
posttest	9.0 (6.9 – 9.4)	0.91	9.1 (7.7 – 10.0)	0.91	0.26
ETGUG 12-18 m, (s)					
pretest	8.3 (6.6 – 12.4)	0.043*	7.4 (6.0 – 9.0)	0.043*	0.347
posttest	8.1 (6.0 – 9.3)	0.91	8.2 (7.9 – 11.0)	0.91	0.30
ETGUG 18-20 m, (s)					
pretest	6.8 (5.9 – 9.7)	0.345	6.5 (5.5 – 8.4)	0.025*	0.917
posttest	6.8 (5.5 – 8.4)	0.42	6.8 (6.3 – 8.4)	0.54	0.03
ETGUG total time, (s)					
pretest	38.9 (30.8 – 52.6)	0.043*	34.1 (28.3 – 38.5)	0.043*	0.076
posttest	35.2 (28.3 – 28.9)	0.91	37.8 (35.8 – 44.1)	0.91	0.56
IMVC left, N					
pretest	293 (63 –698)	0.138	210 (137 – 522)	0.345	0.251
posttest	210 (136 – 793)	0.66	166 (31 – 1032)	0.42	0.36
IMVC right, N					
pretest	241 (67.4 – 595)	0.043*	241 (130 – 711)	0.80	0.465
posttest	282 (75 – 752)	0.91	184 (63 – 521)	0.79	0.23
IRFD left, N/s					
pretest	504 (185 – 1131)	0.043*	417 (130 – 3074)	0.080	0.016*
posttest	937 (631 – 2120)	0.91	67 (35 – 384)	0.79	0.76
IRFD right, N/s					
pretest	325 (138 – 816)	0.043*	417 (130 – 3074)	0.068	0.028*
posttest	862 (617 – 2405)	0.91	212 (112 – 505)	0.82	0.68

Median and interquartile range (IQR) values are at baseline (pre) and after intervention (post). *P*-values after pretest and posttest intervention were computed using the Wilcoxon signed ranks test within intervention and Mann–Whitney U tests for SR-WBV intervention and sham-intervention

*SBBP* Short Physical performance Battery, *ETGUG* Expanded Timed Get Up-and-Go, *ss* sit to stand movement, *IMVC* isometric maximum voluntary contraction, *IRFD* isometric rate of force development, *ES* effect size, *s* seconds, *N/s* Newton/seconds

^*^Statistically significant difference (*p* < 0.05) after pre and post intervention

### Discussing the findings

The present pilot study aimed to develop and test a SR-WBV exercise intervention in LTC. The main focus of this study was to evaluate the feasibility of the SR-WBV intervention and the ability to recruit and retain LTC elderly, and to assess the effects of the intervention. Both the aging population and the number of institutionalized older people are expected to increase in the future. Clinical research in long term care is, however, rather still scarce. Research is, therefore, essential to improve the quality of care in LTC homes. Quality of care and improvement thereof in LTC for the aged is, amongst others, relient on future evidence from research projects and their feasibility in real life conditions.

We demonstrated the feasibility of acquiring acceptable recruitment and compliance rates for LTC dwelling older people randomised in this clinical trial. Our targets of 33 recruitment and 70 % compliance of those training were attained; e.g. those remaining in the study showed excellent compliance with the exercise intervention and retesting. Compared with median rates for recruitment and adherence in falls prevention interventions in institutional settings for clinical trials [12] we achieved better or similar rates for these measures. However, our trial suffered from a rather high attrition rate which we mainly attribute to motivational aspects, e.g. seventeen individuals reported to be not interested or motivated to either begin with exercising or continue to do so. Illness and personal reasons for withdrawal were only explaining training discontinuation of two individuals. Five of initially nine individuals completed the training programme and retest data were obtained from these individuals. Thus, **o**ur trial protocoll in its current form is deemed not feasible in LTC because of the high attrition rate. A future study should aim for an attrition rate of around 15 % [12].

From previous studies we know that the presence of a professional exercise instructor working in the facility was significantly associated with exercise participation and with higher exercise frequencies and levels, and session duration [4]. Furthermore, programs must be carefully designed and coached in order to prevent attrition [19] and be focused on the motivation performing functional activities in the LTC setting [20]. Fairhall et al. [21] postulated that frail elderly individuals should be encouraged and supported to adhere to an intervention plan and studies show higher compliance and fewer dropouts for exercise when the program is accompanied by Motivation-Volition (MoVo) programs [22].

Although we are aware of the fact that the emphasis of a pilot study should be placed on feasibility and not on statistical significance [11] our data allow creating a sample size table for various values of the effect or variance estimates to acknowledge the uncertainty surrounding the pilot estimates. For example, based on an estimated meaningful change in SPPB score of 1 point [23], a significance level set at 5 %, a power of 80 % to detect differences with two-sided hypothesis testing, inclusion of *N* = 30 participants (*n* = 15 per group) will be needed for a future two-groups pre- post-test study design. It should be stressed, however, that this sample size calculation should be interpreted with caution because our estimates may be unrealistic or biased because of the limited sample size [11].

This pilot study provided useful information about the feasibility of the experimental intervention that used SR-WBV for training. Our subjects tolerated the weekly physical intervention well. Those compliant to training were able to progress in intensity and duration of the exercises. Furthermore, our experience suggests that our SR-WBV was of sufficient duration and/or intensity to ameliorate muscle capacity as indicated by improvements in the secondary outcomes. Pragmatically, however, our experimental subjects did not keep up their motivation to perform regular training three times a week, nor were they willing to do so. We believe, therefore, that it is necessary to proceed to an additional study in LTC, however, with major modifications to the protocol. The modifications should thereby focus on improving the motivation to train in LTC dwelling individuals and also assess benefits or risks [24] of this type of training for older people.

Summarising the findings and limitations of this study it becomes clear that this study only reveals first estimates for the chosen outcome measures. We implemented a strict study design to control threats to validity. A necessary next step would be to adapt the study protocol by adding an intervention component that emphasises motivational aspects of exercising and, thus, strive to improve attrition rates in a new LTC SR-WBV exercise group study design as an additional control procedure.

### Conclusions

We conclude that pilot studies with explicit feasibility objectives and success criteria are important foundation steps in preparing for large trials [11] and for development of rehabilitation research programs. Ongoing formal review of the multifaceted issues inherent in the design and conduct of pilot studies can provide invaluable feasibility and scientific data for rehabilitation specialists working in LTC, e.g. physiotherapists, willing to perform clinical trials [25], and may also be highly relevant for furthering the development of theory based rehabilitation [26]. SR-WBV training as applied in this study is deemed only conditionally feasible; it requires some major modifications to the protocol. However, SR-WBV shows trends to stronger improvement in lower extremity muscle properties when compared with sham training. This study encourages the further development of this intervention, preferably with a randomized control design. Future programs must be carefully designed and coached by professional exercise instructors in order to prevent attrition.

## Availability of supporting data

The data set(s) supporting the results of this article are included within the article (and its additional file(s)).

## Additional file

Additional file 1:
**Description of the demografic variables of the participants included in the analysis.** (DOC 31 kb)

## Abbrevations

ES: Effect size
ETGUG: Expanded Timed Get Up-and-Go
IMVC: Isometric voluntary contraction IRFD, Isometric rate of force development
MMSE: Mini-Mental Status Examination
MoVo: Motivation-Volition program
PA: Physical activity
SR-WBV: Stochastic resonance whole-body vibration
WBV: Whole-body vibration
