# Supplementary material for: Preliminary inconclusive results of a randomised double blinded cross-over pilot trial in long-term-care dwelling elderly assessing the feasibility of stochastic resonance whole-body vibration
Source: Eur Rev Aging Phys Act. 2015 Oct 9;12:5. doi: 10.1186/s11556-015-0150-y (PMC4745146; doi:10.1186/s11556-015-0150-y)
Supplement: Additional file 1: — Description of the demografic variables of the participants included in the analysis. (DOC 31 kb) [file 11556_2015_150_MOESM1_ESM.doc]

| **Participant** | **Age** | **Gender** | **Heigth (cm)** | **Weigth (kg)** | **BMI (kg/m^2^)** | **Walking aids** |
| --- | --- | --- | --- | --- | --- | --- |
| 1 | 89 | male | 169 | 78.7 | 27.6 | walking frame |
| 2 | 88 | female | 164 | 59.0 | 21.9 | walking frame |
| 3 | 82 | female | 152 | 73.4 | 31.8 | walking frame |
| 4 | 91 | female | 162 | 57.2 | 21.8 | walking frame |
| 5 | 77 | male | 160 | 96.1 | 37.5 | walking frame |
